# How do short‐term practice effects compare to biomarkers of Alzheimer’s disease?

**DOI:** 10.1002/alz.086315

**Published:** 2025-01-03

**Authors:** Kevin Duff, Dustin B. Hammers, Vincent Koppelmans, Jace B King, John M. Hoffman

**Affiliations:** ^1^ Oregon Health & Science University, Portland, OR USA; ^2^ Indiana University School of Medicine, Indianapolis, IN USA; ^3^ University of Utah, Salt Lake City, UT USA; ^4^ Huntsman Cancer Institute, University of Utah, Salt Lake City, UT USA

## Abstract

**Background:**

Practice effects on cognitive testing in Mild Cognitive Impairment (MCI) and Alzheimer’s disease (AD) remain understudied, especially with how they compare to biomarkers of AD. The current study sought to add to this growing literature.

**Method:**

Cognitively intact older adults (n = 68), those with amnestic MCI (n = 52), and those with mild AD (n = 45) repeated a brief battery of cognitive tests twice across one week, and they also completed a baseline amyloid PET scan, a baseline MRI, and a baseline blood draw to obtain APOE e4 status.

**Result:**

The intact participants showed significantly larger practice effects than the other two groups on an overall composite measure, and those with MCI showed significantly larger practice effects than those with AD. For amyloid deposition, the intact participants had significantly less tracer uptake, whereas MCI and AD participants were comparable. For total hippocampal volumes, all three groups were significantly different in the expected direction (intact>MCI>AD). For APOE e4, the intact had significantly fewer copies of e4 than MCI and AD. Overall, the effect sizes of practice effects were significantly larger than effect sizes of biomarkers in 7 of the 9 comparisons (see Table 1).

**Conclusion:**

Short‐term practice effects on cognitive tests appear to be a sensitive marker in late life cognitive disorders, as they separated groups better than commonly‐used biomarkers in AD. Further refinement of short‐term practice effects as a tool for clinical diagnosis, prognostic indication, and enrichment of clinical trials seems warranted.

 [Table alz086315-tbl-0001]

**TABLE 1 alz086315-tbl-0001:** Effect sizes for markers of Alzheimer's disease.

Marker comparison	Intact vs. MCI	Intact vs. AD	MCI vs. AD
PE vs. composite SUVR	2.20 vs. 1.89, 0.98, 0.33	4.15 vs. 2.02, 5.19, <0.01	1.24 vs. 0.05, 3.93, <0.01
PE vs. hippocampal volume	2.20 vs. 0.87, 4.29, <0.01	4.15 vs. 1.40, 6.84, <0.01	1.24 vs. 0.72, 1.68, 0.09
PE vs. APOE	2.20 vs. 0.98, 4.01, <0.01	4.15 vs. 1.11, 7.71, <0.01	1.24 vs. 0.14, 3.66, <0.01

Note. In each cell is the effect size (i.e., Cohen's d) for practice effects vs. another marker, the z‐value comparing those two effects sizes, and the p‐value of that comparison. PE = practice effects via the standardized‐based regression of Hammers et al. (2021). SUVR = standardized uptake value ratio. MCI = Mild Cognitive Impairment. AD = Alzheimer's disease.

